# An efficient strategy for generation of transgenic mice by lentiviral transduction of male germline stem cells *in vivo*

**DOI:** 10.1186/s40104-015-0058-4

**Published:** 2015-12-24

**Authors:** Jinzhou Qin, Haixia Xu, Pengfei Zhang, Conghui Zhang, Zhendong Zhu, Rongfeng Qu, Yuwei Qin, Wenxian Zeng

**Affiliations:** College of Animal Science and Technology, Northwest A&F University, No. 22 Xinong Road, Yangling, Shaanxi 712100 China

**Keywords:** *In vivo*, Lentivirus vectors, Male germline stem cells, Transgenesis

## Abstract

**Background:**

Male germline stem cells (MGSCs) are a subpopulation of germ cells in the testis tissue. MGSCs are capable of differentiation into spermatozoa and thus are perfect targets for genomic manipulation to generate transgenic animals.

**Method:**

The present study was to optimize a protocol of production of transgenic mice through transduction of MGSCs *in vivo* using lentiviral-based vectors. The recombinant lentiviral vectors with either EF-1 or CMV promoter to drive the expression of enhanced green fluorescent protein (eGFP) transgene were injected into seminiferous tubules or inter-tubular space of 7-day-old and 28-day-old mouse testes. At 5 or 6 wk post-surgery, these pre-founders were mated with wild-type C57BL/6J female mice (1.5 to 2.0-month-old).

**Results:**

Sixty-seven percent of F1 generation and 55.56 % of F2 offspring were positive for eGFP transgene under the control of EF-1 promoter via PCR analysis. The transgenic pups were generated in an injection site-and age-independent manner. The expression of transgene was displayed in the progeny derived from lentiviral vector containing CMV promoter to drive transgene, but it was silenced or undetectable in the offspring derived from lentiviral vector with transgene under EF-1 promoter. The methylation level of gDNA in the promoter region of transgene was much higher in the samples derived lentiviral vectors with EF-1 promoter than that with CMV promoter, suggesting eGFP transgene was suppressed by DNA methylation *in vivo*.

**Conclusion:**

This research reported here an effective strategy for generation of transgenic mice through transduction of MGSCs *in vivo* using lentivirus vectors with specific promoters, and the transgenic offspring were obtained in an injection site-and age-independent manner. This protocol could be applied to other animal species, leading to advancement of animal transgenesis in agricultural and biomedical fields.

**Electronic supplementary material:**

The online version of this article (doi:10.1186/s40104-015-0058-4) contains supplementary material, which is available to authorized users.

## Background

Transgenic animals have been widely used in biomedical, agricultural and veterinary research. There has been increasing interest in developing approaches to modify genome for transgenesis. The techniques, such as pronuclear DNA microinjection [1], embryo stem cell based genome manipulation [2–4], combination of gene modification with nuclear transfer [5, 6] DNA transposon-mediated approaches by microinjection [7], and the gene targeting tools (zinc finger nucleases and TALENs, [8, 9]) have been applied for generating transgenic animals. However, these approaches are complicated, time-consuming, and high cost as well. In recent years, CRISPR-Cas9 system has been widely used for transgenic animals, however it may cause off-target effects *in vivo* [10]. The novel CRISPR/Cpf1 system was efficient for genome-editing in human cells, but has not been used for generating of transgenic animals [11]. Therefore, a simple strategy of transgenesis is highly desirable.

Mammalian spermatogenesis is a continuous, complex process by which spermatogonia proceed through mitosis, meiosis and cytological transformations resulting in formation of numerous spermatozoa throughout the adult life of a male [12]. This spermatogenic process is relied on a subpopulation of germ cells, which are capable of self-renewal and differentiation to spermatozoa, thereby giving rise to the entire spermatogenic lineage. These male germline stem cells (MGSCs) are the only cells in the adult male body that pass the genetic information on to the next generation, making them attractive targets for genetic manipulation [13, 14].

MGSCs based gene modification has comparative advantage over zygote- or oocyte- mediated transgenesis which requires large number of females for super ovulation and expertise for embryo manipulation [15]. Recently, Sahagal and his colleagues [16] reported that manipulation of germ cells through injection of lentiviruses into testis tissue led to generation of transgenic pups with overall success rate as high as 60 %, moreover, the transgene was heritable. Although expression of eGFP was detected under confocal microscopy in multiple tissues of transgenic pups, image of the pups with GFP fluorescence was not presented in their article. The objective of the present study was to simplify and optimize the approach for generating of genome modified mice via lentivirus transduction *in vivo*. Injection of lentivirus into mouse testis tissue resulted in integration of transgene into host genome in an injection site- and age-independent manner. Expression of eGFP transgene was detected in the offspring derived via injection of lentiviral vectors containing eGFP under cytomegalovirus (CMV) promoter, indicating that lentiviral transduction of MGSCs *in vivo* is a simple, efficient, low labor-intensive approach to produce transgenic mice.

## Methods

### Experimental design

Experiment 1 was to optimize the protocol for generation of transgenic mice by lentiviral transduction of male germline stem cells. The effects of injection sites and age of pre-founder mouse was to be elucidated. Experiment 2 was to detect the effect of the promoter for driving the expression of the transgene.

### Animals

C57BL/6J male mice (aged 7 d and 28 d) and C57BL/6J female mice (aged 1.5 to 2 mo.) were obtained from the Fourth Military Medicine University (Xi’an, China). All mice were supplied with water and chow ad libitum, housed and bred in a sterile environment with the controlled temperature (25 ± 5 °C) and humidity (30–70 %), and 14 h night a day. The day of birth was designated as d 0. All protocols for the experiments were approved by and performed under the guidance of the Institutional Animal Care and Use Committee of the Northwest A&F University. All the treatments were carried out in accordance with relevant guidelines and regulations.

### Viral vectors

Lentiviral vectors as a type of retrovirus that can infect both dividing and non-dividing cells were widely used for introduce transgenes or gene knockdown. The non-dividing or terminally differentiated cells such as neurons, macrophages, hematopoietic stem cells, retinal photoreceptors, and muscle and liver cells can be transduced by lentiviral vectors [17]. The lentiviral vectors containing cytomegalovirus (CMV) or human elongation factor-1 (EF-1) promoters to drive eGFP expression were used in this study. The CMV promoter is a widely used promoter and EF-1 is a constitutive promoter of human origin. The lentivirus with EF-1 promoter driving eGFP was packaged in our lab based on the previous protocols [18], and the lentivirus with CMV promoter driving eGFP was a gift from Dr. Wuzi Dong (College of Animal Science and Technology, Northwest A & F University).

### Injection of lentiviral particles and generation of transgenic mice

In Experiment 1, to test age effect of pre-founder, sixteen male mice aged 7 d (body weight 4 ± 1 g) were randomly allocated to two groups, named A and B. And sixteen males aged 28 d (body weight 14 ± 3 g) were randomly allocated to another two groups, named C and D group.

The 7-day-old mice were anaesthetized by intraperitoneal injections of Avertin (400μL/30 g body weight). An incision of approximate 0.3–0.5 cm in length was made in skin and muscle anterior to the penis with a sterile ophthalmology scissors. With the help of a sterile dressing forceps, the testes were moved out from the enterocoelia or scrotal sac gently and the dorsal fat pad was pulled for easy operation. To elucidate an effect of injection site, the lentivirus particles were re-suspended in PBS buffer (Life Technology) with trypan blue (0.04 %). For Group A/C, lentivirus was injected directly into inter-seminiferous tubular space using a syringe with a needle. For Group B/D, lentivirus was injected into seminiferous tubules via efferent ducts or rete testis under a stereoscopic microscopy as described by Shirohana [19]. The pre-founders were implemented artificial feeding for at least 1 wk, then were fed by a maternal mouse for 2 wk followed by self-help feeding. At 6 wk after injection, these pre-founder mice were mated with wild type females of the same strain (aged 1.5–2 mo).

The 28-day-old mice were injected with a higher dose of Avertin (500 μL/30 g body weight) by intraperitoneal, the inguinal area hairs were removed and iodine was used for clean and disinfectant. In an effort to avoid injury to the penis, a wound from skin to muscle anterior to the penis of approximate 0.5–1.0 cm length was cut on a sterile bench. Lentivirus was injected as described above.

Five microlitters of the lentiviral particles at the concentration of 5.5 × 10^6^ TU/mL were used in each injected injection in of this study for 7-day-old mice, and ten microlitters of the lentiviral particles for older ones. All the pre-funders were named 0001, 0002 and so on. In Experiment 2, based on the results of Experiment 1, 10 μL of lentiviral vectors (5.5 × 10^6^ TU/mL) were injected into inter-tubular space of a C57BL/6 J male mice (aged 28 d, *n* = 3). At 5 wk after injection, these pre-founder mice were mated with wild type females of the same strain (aged 1.5–2 mo).

### Genotyping

At 5 or 6 wk post-surgery, the pre-founder male mice were mated with mature wild-type female mice. At 4 wk post-coitum, the new pups of F1 generation were genotyped by PCR in Experiment 1. The eGFP specific primers (Table 2) were used for genotyping. Only those pups with eGFP specific band were considered as transgenic F1 and were named as 1001, 1002 and so on. In order to test whether the transgene could pass to the next generation, half of F1 generation was mated with each other, and the other half was mated with wild-type for F2 generation. The F2 generation pups were named 2001, 2002 and so on.

### Isolation of Genomic DNA (gDNA)

The tail tips (approximately 0.5 cm in length) from F1 and F2 pups and testis tissue from pre-founders were collected for DNA extraction. Samples were cut into small pieces and lysed for 16 h at 55 °C in a high salt digestion buffer containing 50 mmol/L Tris · HCl, 1 % SDS, 100 mmol/L NaCl, 100 mmol/L EDTA and 1,200 μg/ mL Proteinase K (Tiangeng, China). The lysate was processed for extraction of DNA based on salting-out methods as described [19].

### Polymerase Chain Reactions (PCR) and Reverse Transcriptase Polymerase Chain Reaction (RT-PCR)

Ubiquitous *gapdh* gene was amplified as a loading control. The pCD513B-CMV-MCS-EF1 plasmid DNA was introduced as a positive control while the genomic DNA obtained from wild-type mice as a negative control. RNA was isolated from transgenic F1 and F2 animals with TRIZOL (Life Technology, USA) and reverse transcribed (RT) to cDNA with Superscript III RT First Strand cDNA synthesis Kit (Invitrogen, USA) according to the manufacturer’s protocol. PCR was performed in duplicate in a 20 μL of reaction volume consisting of TaKaRa Ex Taq (TAKARA, Japan), 0.5 μmol/L of each primer and 500 ng of gDNA or cDNA. The PCR protocol included one cycle at 94 °C for 10 min, 32 cycles of denaturation at 95 °C for 15 s, annealing at 60 °C for 30 s and extension at 72 °C for 30 s, then followed final extension for 10 min at 72 °C. The PCR products were visualized after 2 %-agarose gel electrophoresis stained with ethidium bromide (Sigma-Aldrich, USA).

### Western blot

Proteins of the interest tissues (heart, skin, liver, muscle and testis) were isolated from transgenic mice that were confirmed by RT-PCR (non-transgenic pups as negative control). The protein concentration was determined using a Quick Start™ Bradford Protein Assay Kit (Bio-Rad, USA). Protein complexes were separated by SDS-PAGE, and transferred to nitrocellulose membranes (Hybond ECL, USA). Membranes were probed using the following primary antibodies: anti-β-actin (Abcom, 1: 1,000), anti-GFP (Abcom, 1: 1,000). Secondary antibodies were horseradish peroxidase-linked anti-rabbit antibody (Santa Cruz, 1: 2,000). Protein bands were visualized on a Bio-Rad Chemidoc XRS using a Western Bright ECL Kit (Advansta, Menlo Park, CA, USA).

### Histology

The testis tissue from transgenic mice (aged 2 mo) in Experiment 2 was collected and fixed in Bouin’s solution overnight, embedded in paraffin for serial sectioning at 5–7 μm. The sections were stained with hematoxylin and eosin (H.E., Sigma-Aldrich, USA) and viewed under the light microscope (Olympus, Japan).

### Bisulfite sequencing PCR

The genomic DNA from the testis tissue of transgenic F1 mice (pre-founder transduced with lentivirus with promoter CMV or EF-1) was directly subjected to bisulfite conversion with EZ DNA Methylation Direct kit (Zymo Research, Orange, CA, USA) according to the manufacturer's protocol. The BSP specific primers (Table 2) were used for bisulfite-modified DNA amplification. After bisulfite conversion, the PCR products were cloned into a pGEM-T Easy Vector (NEB, England), and nine individual clones with different promoters were sequenced by BIO5 Institute, University of Arizona.

## Results

To compare an effect of site at which the lentivirus was injected, recombinant lentiviral vectors (pCD513B-CMV-MCS-EF1) containing an EF-1 promoter to drive eGFP expression were injected into the inter-tubular space of testis (group A/C) or seminiferous tubules (group B/D). At 5 or 6 wk after injection, pre-founder mice were mated with wild type females of the same strain (1.5 to 2.0-month-old), as it needs around 35 d to fulfill the entire process of spermatogenesis in mice [20].

**Fig. 1:**
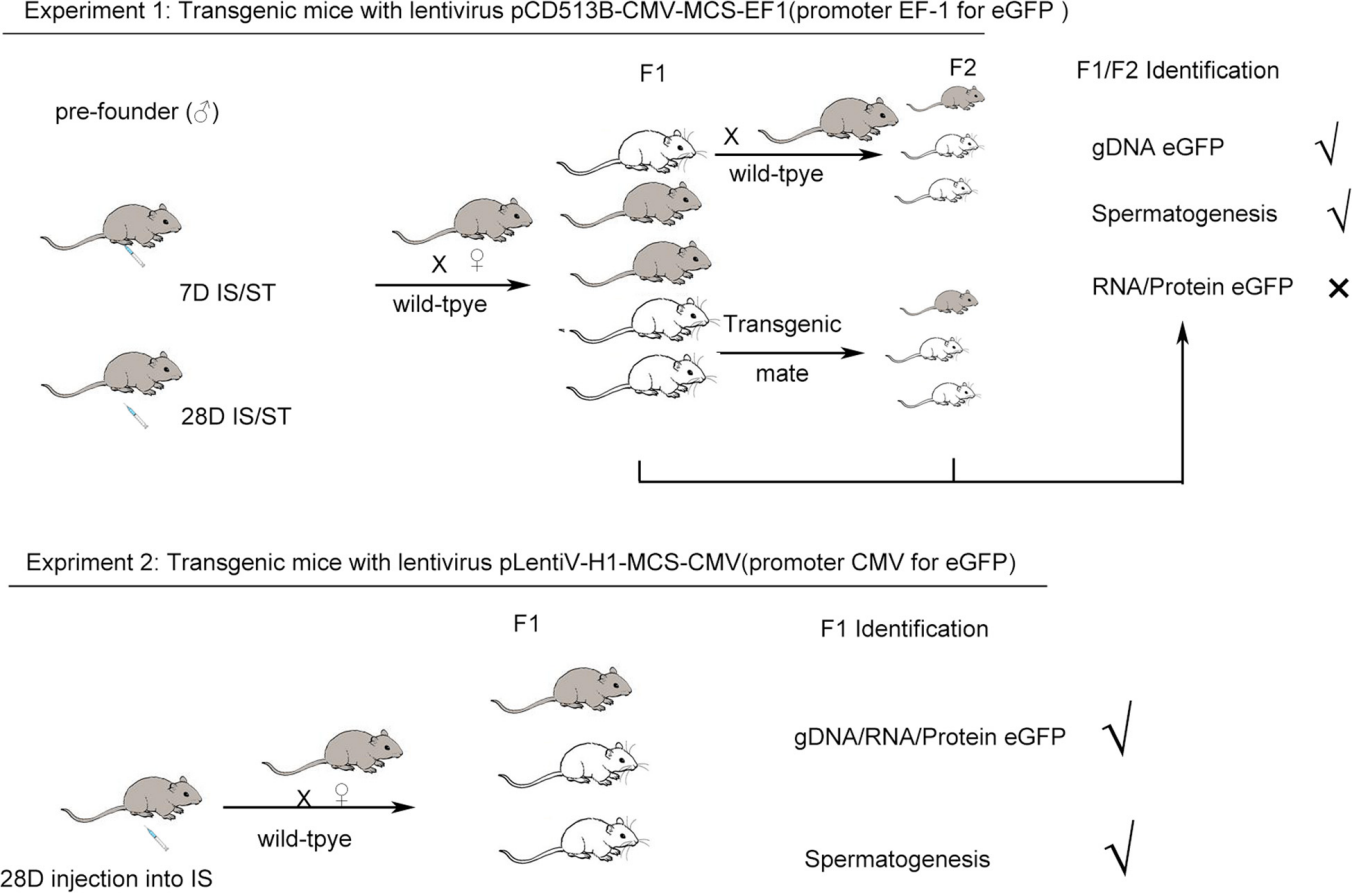
A schematic representation of procedures involved generation of transgenic mice by manipulation of MGSCs *in vivo* with recombinant lentivirus. IS: injection of lentivirus into inter-tubular spaces; ST: injection of lentivirus into seminiferous tubules

**Fig. 2:**
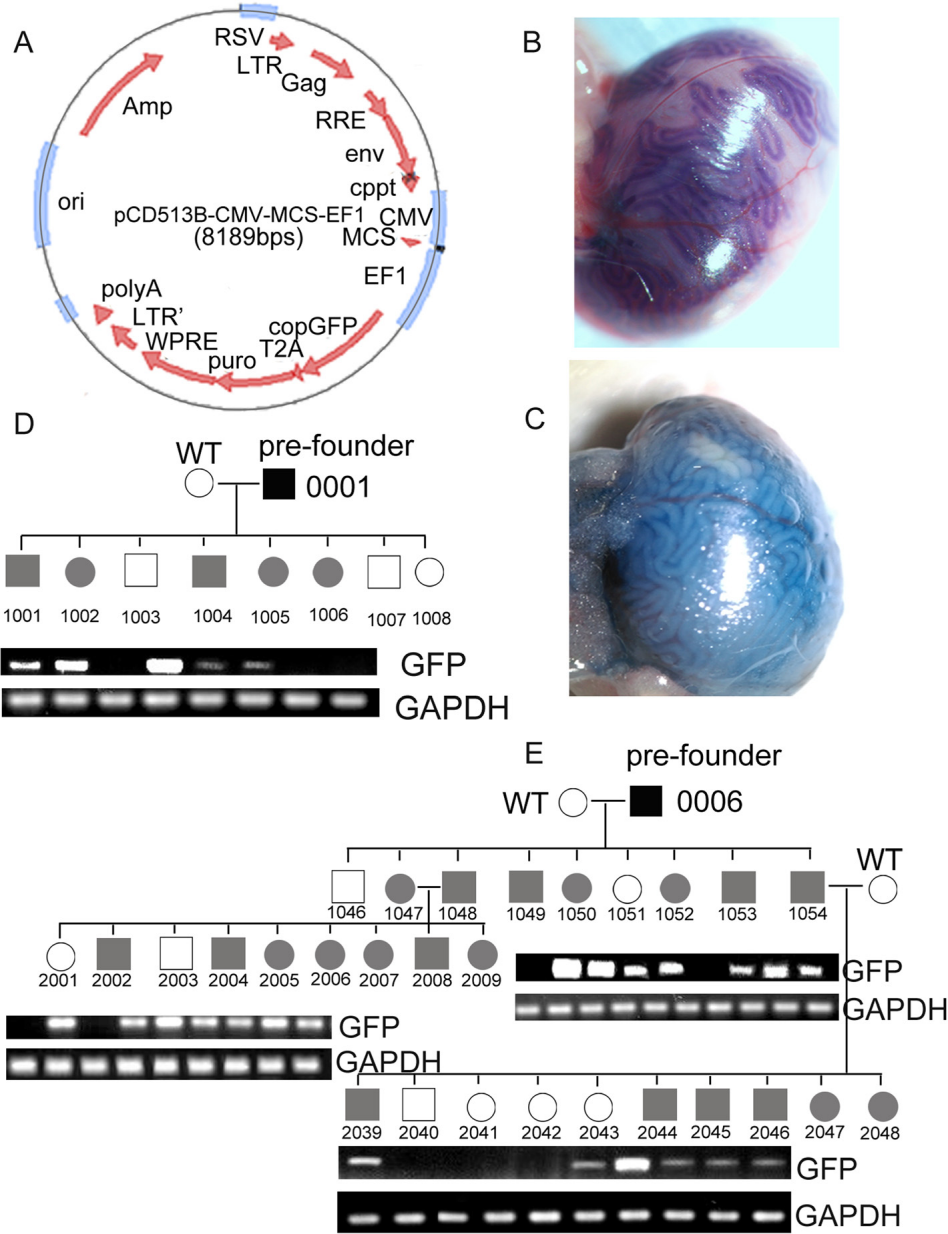
Generation of transgenic mice via injection of lentivirus with EF1 promoter to drive eGFP expression in Experiment 1. **a**. Lentiviral vector used for generating transgenic mice. **b-c**. Recombinant lentiviruses injection into mouse testis via seminiferous tubules and inter-tubular spaces. **d-e**. Phenotype identification showing all germline transmission of the transgene such as pedigree analysis from pre-founder mice 006 (**e**) and 001 (**d**) with different ages or injection areas. gDNA amplification using the primers for eGFP transgene or GAPDH as shown

To test the *eGFP* transgene in pre-founder testis and F1 pups, eGFP specific primers and *gapdh* primers (as a loading control) were used for DNA amplification (Table 1). To our great surprise, overall 67.88 % (131/193) of F1 pups were positive for eGFP transgene (Fig. 2d–e, Table 1 and Additional file 1: Table S1). Interestingly, there was no significant difference in F1 transgenic rate either between two injection sites or between two ages of pre-founders (*P* > 0.05). F2 generation was generated from either transgenic F1 animals mating each other or transgenic F1 mice mating with wild-type C57BL/J mice (Fig. 1). The *eGFP* gene can be identified from these two strategies in F2 pups with transgenic rate over 55.0 %, indicating the transgene could be heritable. The transmission rate was as high as 55.6 % in F2 pups (Fig. 2, Table 1 and Additional file 1: Table S1). However, the transgene expression was not detectable under a fluorescence microscopy or via RT-PCR.

**Table 1:** Percentage of eGFP positive pups obtained from F1 and F2 generations in Experiment1

Genetarion	Groups		No. litters	No. positive pups	Percentage
F1 generation	Pre-founder age	Injection methods			Success rate, %
	7 d	IS group A	31	21	67.8
		ST group B	60	40	66.7
	28 d	IS group C	45	30	66.7
		ST group D	57	40	70.2
	Grand total		193	131	67.88
F2 generation	Mating				Transmission rate, %
	Transgenic × Wild type		15	7	46.78
	Transgenic × Transgenic		21	13	61.90
	Grand total		36	20	55.56

*IS* injection of lentivirus into inter-tubular spaces, *ST* injection of lentivirus into seminiferous tubules

The above observations from Experiment 1 suggested that the transgene was silenced or its expression was undetectable. As there was no difference for transgenesis between two injection sites, in Experiment 2, lentivirus (pLentiV-H1-MCS-CMV) with CMV promoter driving eGFP was injected into the inter-tubular space of mouse testes. The pre-founder preparation and F1 generation were implemented as the protocol described as Experiment 1 (shown in Fig. 1). Total ten pups of F1 generation were obtained from two pre-founders.

**Fig. 3:**
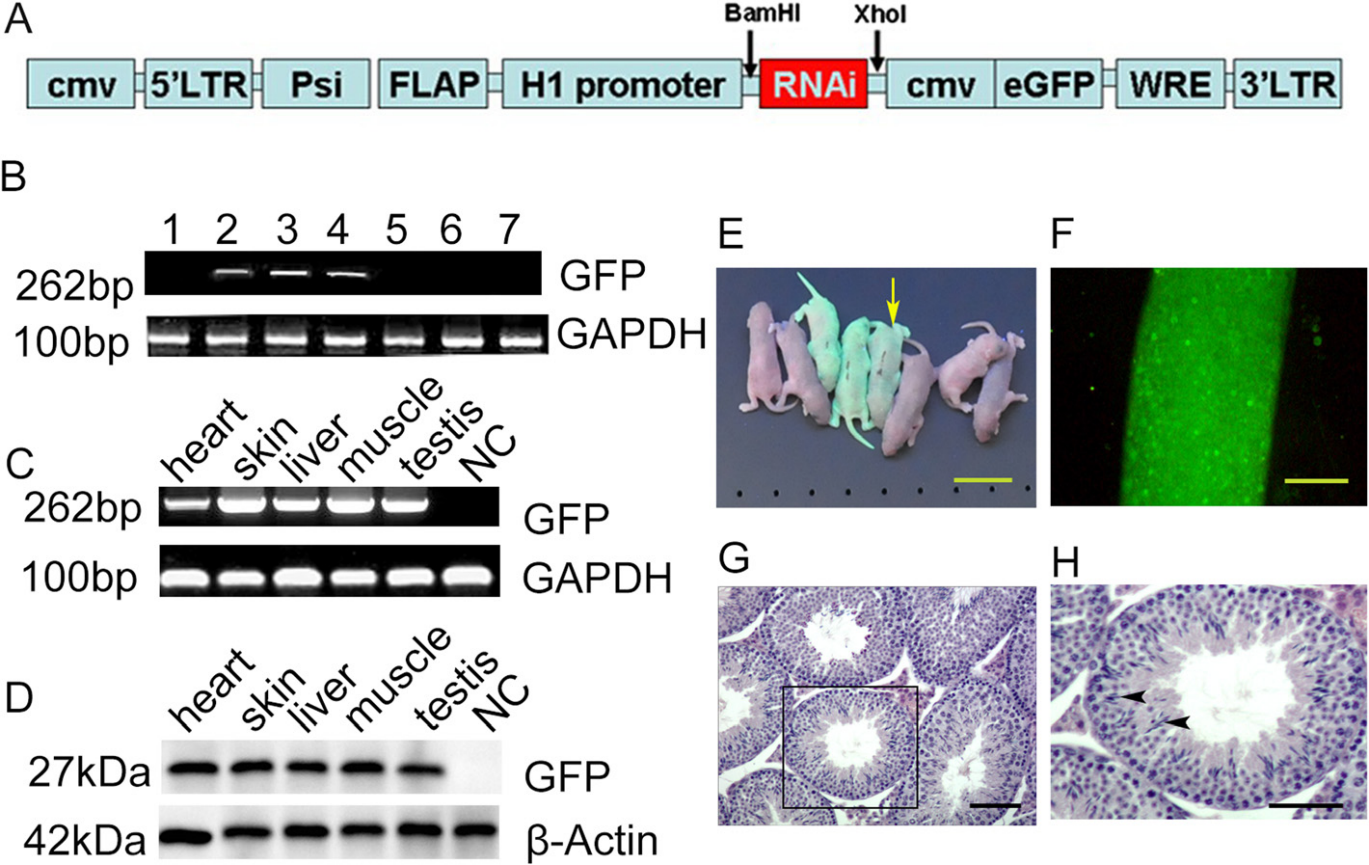
Generation of transgenic mice via injection of lentivirus with CMV promoter to drive eGFP expression in Experiment 2. **a**. Lentiviral vector used for generating transgenic mice. **b**. gDNA amplification using the primers for eGFP transgene or GAPDH. Heart, skin, liver, muscle and testis tissue were collected from a pup indicated in (**e**), and the expression of eGFP transgene was detected via RT-PCR (**c**), Western Blot (**d**). E. eGFP expression in F1 pups was observed under a fluorescent lamp. F. Expression of eGFP in a seminiferous tubule. G. A cross section of testis tissue from a F1 transgenic mouse showed complete spermatogenesis. Bar = 100 μm in F, G and H, Bar = 1 cm in E

DNA from the tail samples of the pups was extracted for PCR. Three samples from one litter displayed *eGFP* positive bands (Fig. 3b). *gapdh*, a housekeeping gene, was served as a loading control. Under a fluorescence lamp, five of the ten pups showed noticeable green fluorescence. RT-PCR and Western Blot analysis further confirmed the expression of eGFP transgene in different tissues of the No.3 pup (Fig. 3e). A non-transgenic testis was as negative control (NC) (Fig. 3c and d). Three of seven F1 pups in one litter showed eGFP fluorescence (Fig. 3e), and two of three F1 pups in another litter showed eGFP fluorescence. After dissection, seminiferous tubule from an adult transgenic F1 generation showed eGFP fluorescence under fluorescence microscope (Fig. 3f). The cross section of testis tissue showed the complete spermatogenesis with spermatozoa (Fig. 3g, h). As CMV promoter may drive eGFP expression in both somatic Sertoli cells and germ cells within seminiferous tubules, to further confirm whether germ cells were indeed expressed eGFP transgene, immunohistochemistry was conducted using germ cell specific marker (VASA). As shown in Additional file 1: Figure S2, some eGFP green cells were expressed VASA, suggesting that eGFP transgene was expressed in germ cells and thus may passage to next generations.

**Fig. 4:**
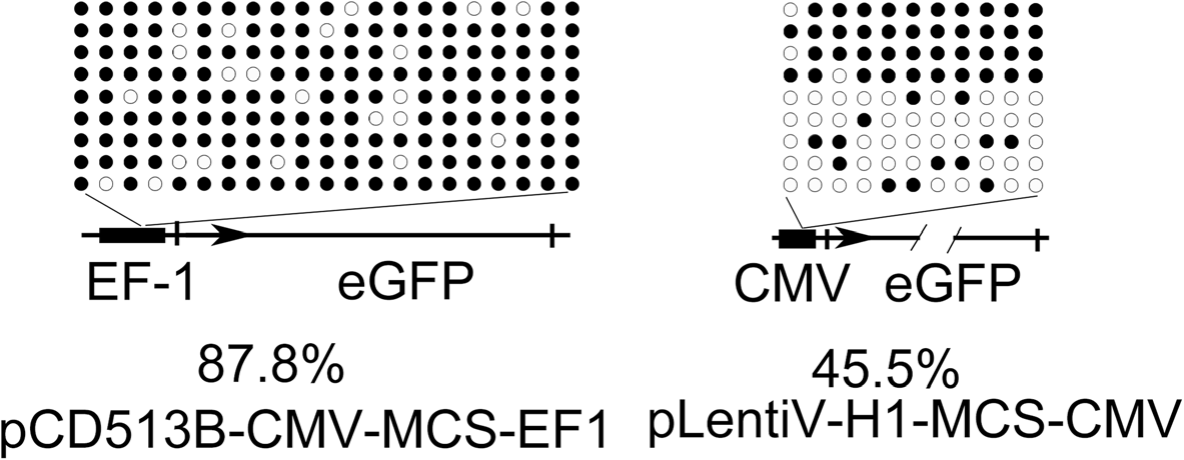
Analysis of DNA methylation in the promoter of *eGFP* gene. The analysed position of CpG dinucleotide showed by a horizontal line. Open circles, unmethylated CpG; closed circles, methylated CpG

To further elucidate whether the silence of GFP transgene was related with DNA methylation at the promoter region, which was used to drive the gene of interest, DNA methylation level in the EF-1 and CMV promoters were analyzed. DNA from the lentiviral-medicated F1 generation was subject to bisulfite convention. Specific primers for bisulfite sequencing PCR (BSP, Table 2) were used for amplifying DNA methylation in the region of EF-1 or CMV promoter. DNA methylation in EF1 and CMV promoter were totally different, 87.8 % vs. 45.5 % (Fig. 4), suggesting that DNA from the pCD513B-CMV-MCS-EF1 lentivirus-mediated transgenic pups (Experiment 1) displayed much higher methylation level in the region of promoter for driving eGFP expression than that from pLentiV-H1-MCS-CMV lentivirus-mediated transgenic mice (Experiment 2). These data indicates that expression of eGFP in Experiment 1 was repressed by DNA methylation.

**Table 2:** Specific primers used in this study

Name of oligonucleotide	Sequence
eGFP Forward	GACGTAAACGGCCACAAGTT
eGFP Reverse	TCTTGTAGTTGCCGTCGTCC
GAPDH Forward	CTCTCTGCTCCTCCCTGTTCC
GAPDH Reverse	CCAAATCCGTTCACACCGACC
BSP CMV Forward	TAAAAATAAATTATAAAAATTTAAAATTTT
BSP CMV Reverse	AATACCAAAACAAACTCCCATTAAC
BSP EF-1 Forward	TGTTTGATTTTGTTTGTTTAATTTTA
BSP EF-1 Reverse	ACCCTACTTAAAAATACCCTCTCC

The above data provided an efficient, quick and effective protocol to generate transgenic mice via transduction of MGSCs with lentiviral vectors *in vivo*. This strategy did not compromise the fertility and development of descendants, resulting in transmission of the transgene through the male germ line.

## Discussion

Recently, Sehgal et al. [16] reported that injection of lentiviral vector into mouse testes resulted in transduction of spermatogonia, and the pre-founder male mice could sire and produce transgenic pups. However, images of eGFP expressing pups were not shown in their article. Effects of injection techniques and age of on the efficiency of transgenesis were further conducted in the present study. Moreover, the differences in DNA methylation of two different promoters for driving transgene expression were analyzed. We found that transgenic mouse could produce in an injection site-and age-independent manner through transduction of MGSCs *in vivo* using lentivirus vectors with specific promoters. Transgenic pups with eGFP fluorescence were generated using lentiviral vector in which eGFP expression cassette was under the control of CMV promoter.

Mammalian spermatozoa are produced in the seminiferous tubules which are compacted inside of testis. It is not known whether injection of lentivirus into the lumen of seminiferous tubules would lead to higher efficiency of transgenesis, compared to inter-seminiferous tubule injection. Therefore, first, we compared the strategies for injection of lentivirus. Similarly as the technique of germ cell transplantation, lentiviral based-vector was injected into seminiferous tubules via either rete testis or efferent ducts. Another method is that lentivirus was directly injected into inter-tubule space. We found that there was no significant difference for transgenesis between these two injection methods. Either microinjection into seminiferous tubules or direct injection into inter-tubular space transduced MGSCs with lentiviral vectors. Therefore, the simple and easily direct injection method was used in Experiment 2.

Second, gonocytes are the only type of germ cells in new-born mouse seminiferous tubules. By seven d after birth, gonocytes are migrated toward and attached to the basement membrane of seminiferous tubules and have differentiated to form type A spermatogonia among which undifferentiated spermatogonia are regarded as spermarogonial stem cells (SSCs), which were a sub-population of MGSCs [21]. By 28 d after birth, different types of germ cells are arranged in the epithelia of seminiferous tubules in which MGSCs are localized at the basement membrane. The rate of SSCs is higher in neonatal testis tissue than that in 28-day-old one. It is unclear whether infection of male germ cells *in vivo* at 7-day-old age would higher efficiency than 28-day-old age. Therefore, effect of pre-founder on the transgenic efficiency was compared in our study. We found that efficiency of eGFP transgenesis between these two ages of pre-founders (Table 1). Therefore, these observations indicate that lentiviral vectors could successfully delivery genetic information to MGSCs in an injection site- and age-independent manner. As far as we know,it is the first time to present microinjection of virus into neonatal testis for production of transgenic animals.

Lentiviral vectors have been an attractive tool for transgensis because of their ability to transduce both dividing and quiescent cells and the high integrating efficiency [17, 22]. Meanwhile, male germline stem cells (MGSCs) are perfect targets for transgenesis as they are the foundation of spermatogenesis [23]. Thus, a few studies have examined the utilization of lentivirual vectors as a tool to modify MGSCs genome in mice [24], rats [25–27] and pig [28, 29]. Nagano et al. [24] found that transplantation of the lentiviral vector-modified mouse MGSCs resulted in the establishment of spermatogenic colonies in all of the recipient mouse testis, but the authors did not examined the transmission of transgene in the subsequent generation. Hamra et al. [27] reported that 59 % of the rat offspring were transgenic when the recipients of transplantation of lentiviral particle-mediated rat MGSCs were mated wild type females. Meanwhile, Ryu et al. [26] also found that rat MGSCs were transduced efficiently with lentiviral particles, which led to the production of transgenic progeny following transplantation of the transduced MGSCs and mating of the recipient males. In a more recent study, Kanatsu-Shinohara et al. [25] observed that transduced rat MGSCs produced eGFP transgene-expressing spermatogenic cells when lentiviral vector-mediated cells were xeno-transplanted into the seminiferous tubules of immunodeficient mice, and microinsemination of these transgenic germ cells resulted in stably transmitted the eGFP transgene in the next generation. Lately, Zeng et al. [28] reported that lentivirus-based vector was effective in transducing pig MGSCs, resulting in the production of eGFP transgenic spermatozoa in recipient boars. Semen collected from these boars generated 21 % transgenic IVF embryos, indicating that lentivirus-mediated MGSCs transduction results in transgene transmission in pigs as well. Thus combining of transplantation with transduction of MGSCs using lentivirus-based vectors can generate transgenic animals.

However, multiple factors contribute the overall low efficacy of generating transgenic animals by transducing MGSCs followed by transplantation. Preparation of recipient animals for transplantation is a big challenge. Inappropriate recipient preparation would lead to inefficient colonization of donor MGSCs and low fertility of the recipients. The amount of donor-derived spermatozoon production relative to endogenous production in the recipient host testis is another challenge, especially in rats and large animals. Therefore, alternative strategy for transgenesis is desirable.

The promoter affects the expression of transgene. A few studies showed that lentivirus particles could effectively transduce MGSCs in vitro and *in vivo*, and transgenic animals expressed GFP transgene under the control of EF-1 promoter [15, 30, 31]. Nagano et al. [24]reported that CMV promoter was not effective in lentiviral transduction and transgene expression in MGSCs. However, recently Liu et al. [32] and Usmani et al. [15] demonstrated that CMV promoter could drive transgene expression as well, which is in consistent with the findings obtained from the present study. As DNA methylation is generally associated with transcriptional silencing [33], we analyzed the methylation level in the region of promoters. The pups generated in Experiment 1 with pCD513B-CMV-MCS-EF1 expressed eGFP reporter gene in neither mRNA level nor protein level. Using BSP analysis, we found that DNA from the pups that were derived from transduction of pCD513B-CMV-MCS-EF1 lentiviral vector displayed much higher level of DNA methylation in the promoter region than that DNA from pLentiV-H1-MCS-CMV, suggesting that the transgene was suppressed by DNA methylation *in vivo*. That is probably the main reason why the eGFP transgene was silenced or its expression was under detectable in Experiment 1. The DNA Methyl-transferase inhibitor should be explored in the research for driving the transgene expression with the specific promoter. Two promoters (EF1 and CMV) for driving eGFP expression were studied in this study. But experiments using testis tissue or germ cell specific promoter was not included in the present study. Lentiviral particles with spermatogonial stem cell (SSC) specific promoter would infect SSC solely in testis and probably lead to transgenic spermatozoa more efficiently, therefore, should be used in the future study. Therefore, a specific promoter of lentiviral vectors should be carefully considered for transgenic generation *in vivo*.

## Conclusions

In conclusion, the strategy demonstrated here a stable, effective and reproductive way to produce transgenic mice with high success rate. Furthermore, this protocol could be applied to other animal species, especially livestock, leading to significant advancement of animal transgenesis in agricultural and biomedical fields.

## Additional file

Additional file 1:
**More information about transgene in Experiment 1 and Experiment 2.** (DOC 812 kb)
