# Supplementary material for: An efficient strategy for generation of transgenic mice by lentiviral transduction of male germline stem cells in vivo
Source: J Anim Sci Biotechnol. 2015 Dec 24;6:59. doi: 10.1186/s40104-015-0058-4 (PMC4690335; doi:10.1186/s40104-015-0058-4)
Supplement: Additional file 1: — More information about transgene in Experiment 1 and Experiment 2. (DOC 812 kb) [file 40104_2015_58_MOESM1_ESM.doc]

Table S1, the statistics of the number of pups and the number of positive pups from individual matings in experiment 1.

| Group | Pre-founder or founder mice | Offspring per litter | Offspring per litter positive for EGFP |
| --- | --- | --- | --- |
| Group A | 0001 X WT ^a^ | 8 | 5 |
|  | 0001 X WT | 9 | 6 |
|  | 0001 X WT | 7 | 6 |
|  | 0001 X WT | 7 | 4 |
| Group B | 0002 X WT | 7 | 5 |
|  | 0002 X WT | 6 | 3 |
|  | 0002 X WT | 6 | 4 |
|  | 0002 X WT | 9 | 5 |
|  | 0003 X WT | 8 | 6 |
|  | 0003 X WT | 6 | 4 |
|  | 0003 X WT | 9 | 8 |
|  | 0003 X WT | 9 | 5 |
| Group C | 0004 X WT | 7 | 6 |
|  | 0004 X WT | 7 | 3 |
|  | 0004 X WT | 6 | 5 |
|  | 0005 X WT | 5 | 4 |
|  | 0005 X WT | 8 | 5 |
|  | 0005 X WT | 7 | 4 |
|  | 0005 X WT | 5 | 3 |
| Group D | 0006 X WT ^b^ | 9 | 7 |
|  | 0006 X WT | 7 | 4 |
|  | 0006 X WT | 8 | 6 |
|  | 0006 X WT | 9 | 6 |
|  | 0007 X WT | 7 | 4 |
|  | 0007 X WT | 9 | 7 |
|  | 0007 X WT | 8 | 6 |
| F2 | 1004 X WT | 5 | 1 |
|  | 1054 X WT ^c^ | 10 | 6 |
|  | 1032 X 1022 | 5 | 2 |
|  | 1027 X 1015 | 7 | 4 |
|  | 1047 X 1048 ^d^ | 9 | 7 |

Note: a, more detail information in Figure 2D (wildtype X pre-founder 0001). b, more detail information in Figure 2E (wildtype X pre-founder 0006). c, more detail information in Figure 2E (wildtype X 1054). d, more detail information in Figure 2E (1047 X 1048). WT: wildtype.

Figure S1


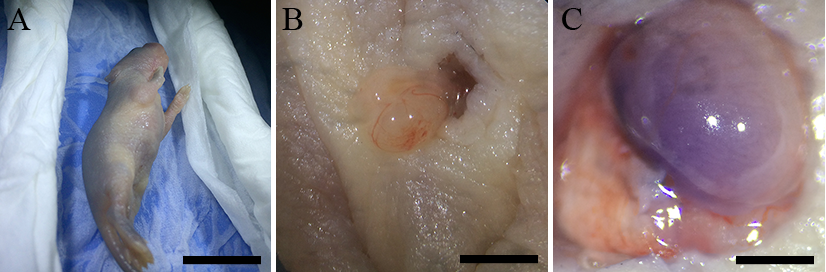


Note: Male germline stem cells transplantation procedure of C57BL/6J mouse (7-days-old). A: Place the anaesthetized C57BL/6J mouse on a sterile bench; B: Expose testes by operation under an anatomical microscope; C: Show the testes with successful injection into the seminiferous tubules.

Bar=1.5cm in A; Bar=0.5cm in B; Bar=0.15cm in C.

Supplementary figure 2


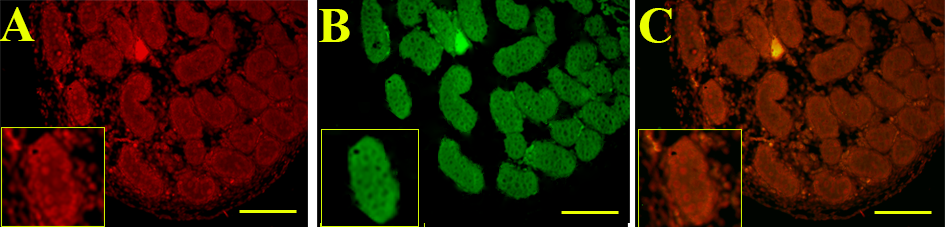


Note: Expression of EGFP and VASA in the testis section. A, a cross section of testis tissue from a F1 transgenic mouse aged 7 days showed EGFP expressed in all cells（Anti-EGFP antibody from goat was used, and dinky-anti-goat secondary antibody conjugated with Texas Red）. B, VASA specific expressed in germ cells. （Anti-VASA antibody from rabbit was used, and dinky-anti-rabbit secondary antibody conjugated with FITC）. C，Merge. Bar=100um in A, B and C.
